# An all-out assault on a dominant resistance gene: Local emergence, establishment, and spread of strains of tomato spotted wilt orthotospovirus (TSWV) that overcome Sw-5b-mediated resistance in fresh market and processing tomatoes in California

**DOI:** 10.1371/journal.pone.0305402

**Published:** 2024-07-10

**Authors:** Mônica A. Macedo, Tomas Melgarejo, Margaret Cespedes, Maria Rojas, Patrícia Lazicki, Thomas Turini, Ozgur Batuman, Robert Gilbertson

**Affiliations:** 1 Federal Institution of Brasília, Brasília, Federal District, Brazil; 2 Department of Plant Pathology, University of California Davis, Davis, California, United States of America; 3 University of California Cooperative Extension, Woodland, California, United States of America; 4 University of California Agriculture and Natural Resources, Fresno, California, United States of America; 5 Department of Plant Pathology, Southwest Florida Research and Education Center, University of Florida, Immokalee, Florida, United States of America; University of Florida Institute of Food and Agricultural Sciences, UNITED STATES

## Abstract

Tomato spotted wilt orthotospovirus (TSWV) causes substantial economic loss to tomato production, and the *Sw-5b* resistance gene is widely deployed for management. Here, we show (i) the emergence of resistance-breaking (RB) TSWV strains in processing and fresh market tomato production in California over the past ten years, and (ii) evolutionary relationships with RB strains from other areas. A specific RT-PCR test was used to show the C118Y RB strain that emerged in Fresno County in 2016 quickly became predominant in the central production area and remained so through this study. In 2021, the C118Y strain was detected in the Northern production area, and was predominant in 2022. However, in 2023, the C118Y strain was unexpectedly detected in fewer spotted wilt samples from resistant varieties. This was due to emergence of the T120N RB strain, previously known to occur in Spain. A specific RT-PCR test was developed and used to show that the T120N RB strain was predominant in Colusa and Sutter counties (detected in 75–80% of samples), and detected in ~50% of samples from Yolo County. Pathogenicity tests confirmed California isolates of the T120N strain infected Sw-5b tomato varieties and induced severe symptoms. Another RB strain, C118F, was associated with spotted wilt samples of *Sw*-5 varieties from fresh market tomato production in southern California. Phylogenetic analyses with complete NSm sequences revealed that the C118Y and T120N RB strains infecting resistant processing tomato in California emerged locally, whereas those from fresh market production were more closely related to isolates from Mexico. Thus, widespread deployment of this single dominant resistance gene in California has driven the local emergence of multiple RB strains in different tomato production areas and types. These results further emphasize the need for ongoing monitoring for RB strains, and identification of sources of resistance to these strains.

## Introduction

Tomato spotted wilt orthotospovirus (TSWV, synonym *Orthotospovirus tomatomaculae*) causes spotted wilt disease of pepper (*Capsicum annuum*), tomato (*Solanum lycopersicum*) and other crops [1]. The disease can cause substantial economic loss in fresh market and processing tomato production and is emerging as a global threat to production of this critical vegetable crop [2]. TSWV is transmitted by various species of thrips, especially the supervector western flower thrips, *Frankliniella occidentalis*. The mode of transmission is persistent propagative, the virus is not transovarially transmitted, and larval forms must acquire the virus for adults to become viruliferous [3, 4]. TSWV is not seed-transmitted in tomato or spread by contact; however, it is not phloem-limited and is mechanically transmissible to some hosts, including pepper and tomato, under laboratory conditions. Effective management of spotted wilt in tomato is best accomplished with an integrated pest management (IPM) approach, which often involves the planting of varieties with the *Sw*-*5b* resistance gene [5].

The *Sw-5b* gene is a single, dominant resistance (R) gene that encodes a protein with nucleotide-binding (NB) and leucine-rich repeat (LRR) domains, typical of the NLR type of R protein, as well as a Solanaceae-specific N-terminal domain (SD) [6–8]. The LRR and SD domains of the Sw*-*5b protein are involved in a dual recognition of the viral effector, the NSm (movement) protein. Recognition leads to R protein activation and triggering of a hypersensitive response (HR), characterized by necrotic local lesions on leaves, which typically appear 2 days after inoculation. Interestingly, the *Sw-5b* gene also confers resistance to other tospoviruses from the New World (NW), e.g., tomato chlorotic spot virus (TCSV), groundnut ringspot virus (GRSV), and chrysanthemum stem necrosis virus (CSNV) [9, 10]. Remarkably, the Sw-5b R protein recognizes the geminivirus C4 effector [11], but it does not confer resistance to infection by geminiviruses. The *Sw-5* gene does not confer resistance to tomato-infecting tospoviruses from the Old World (OW), e.g., groundnut bud necrosis virus, capsicum chlorosis virus and tomato zonate spot virus [12]. The capacity of *Sw-5b* to recognize multiple viruses is unusual for this type of R gene, including the *Tsw* R gene in pepper, which is effective only against certain TSWV strains [13].

The robust resistance provided by the single dominant *Sw-5b* R gene led to relatively rapid introgression into commercial fresh market and processing tomato varieties, which were widely planted in many growing areas, e.g., in countries of Europe, South America, and Asia; in Australia, Canada, Mexico and South Africa; and in many states of the U.S., including California. This in turn has led to the relatively rapid (within ~5 years) emergence of resistance-breaking (RB) strains of TSWV in multiple tomato production areas [14–22]. Analyses of the nucleotide (nt) and amino acid (aa) sequences of NSm genes and proteins of RB strains has revealed a region of ~20 aa that is recognized by the *Sw-5b* R protein [12]. Furthermore, aa changes in this region, e.g., at position 118 to aromatic aas with bulky side chains such as tyrosine (Y), have been associated with RB. These mutations are believed to mask this region from Sw-5b recognition while not interfering with NSm cell-to-cell movement function [12]. The C118Y (C118Y) mutation has been identified in RB isolates from many areas of the world, e.g., South Africa [14], Australia [15], Spain [16], Italy [17], United States [19], Turkey [20], and Serbia [21]. Subsequently, other aa substitution mutations have been identified in this NSm region that also allow for RB, e.g., T120N, C118F, and D122G. The T120N and C118F strains have been exclusively reported from Spain and Mexico, respectively [18, 22]; whereas the D122G strain was reported from Australia, Hungary and the US (North Carolina) [18, 23, 24]. Together, these results demonstrate TSWV is rapidly overcoming Sw-5b resistance, and spotted wilt could reach pandemic levels in areas where varieties with this resistance gene are exclusively grown. In addition to the selection exerted by the widespread deployment of the *Sw-5b* gene, a recent report by Chinnaiah et al. (2023) [25] revealed vector associated selection based on the increased fitness in thrips viruliferous for RB strains compared with those viruliferous for non-RB strains or non-viruliferous. Together, these results help explain how RB strains have spread so rapidly in California.

Here, we document the emergence of multiple RB TSWV strains in processing and fresh market tomato varieties grown in multiple growing areas of California, and show the evolutionary relationships among these and previously described RB strains. For processing tomatoes this involved the statewide monitoring of the C118Y strain detected in Fresno County, California in 2016 and that has caused substantial economic loss to fresh market and processing tomato production in California [19]. Using a RT-PCR test specific for this C118Y strain, we show how it (i) became dominant in the central production area, (ii) spread to the northern production area in 2021, and (iii) interacted with the new T120N strain that emerged in Colusa and Sutter counties in 2023. In contrast, C118F and C118Y strains were detected in spotted wilt samples from resistant fresh market varieties grown in Mexico in 2014 and in southern California and New Jersey in the US from 2021–2023. This indicated that certain RB strains were associated with different geographical areas and type of tomato production, which was in agreement with results of phylogenetic analyses of NSm sequences that showed the C118Y and T120N strains infecting processing tomatoes in California emerged locally, whereas the fresh market C118F and C118Y strains emerged separately and were more closely related to strains from Mexico. Pathogenicity tests showed that these RB strains, including the new T120N strain from northern California, are highly virulent on resistant and susceptible varieties, consistent with how rapidly these strains have emerged, spread and became predominant in California, where Sw-5b resistance varieties are almost exclusively planted. Together, our findings highlight the evolving landscape, adaptation and competition among RB strains in California. This also has created a potential pandemic situation as Sw-5b resistance becomes less effective in more production areas.

## Materials and methods

The emergence of the C118Y strain in Fresno County in 2016 was a major concern because most processing and fresh market tomato varieties planted in the central production area possessed *Sw*-5b resistance. Thus, it was important to assess the survival and potential of this strain to infect resistant processing tomato varieties in subsequent growing seasons in this area. Thus, surveys for plants with representative spotted wilt symptoms in processing tomato fields in California were conducted in July of 2017–2023, and leaf and fruit samples were collected from plants representative wilt symptoms (5-10/field). In addition, spotted wilt samples were received from our statewide network of Farm Advisors, pest control advisers (PCAs) and growers throughout the growing season (Table 1). Samples of tomato plants with spotted wilt symptoms were sent or brought to our laboratory with a general diagnostic permit for such samples (permit #2919) issued by the California Department of Food and Agriculture (CDFA) to R. L. Gilbertson. Permission to enter processing tomato fields was obtained by UCCE Farm Advisor P. Lazicki.

**Table 1:** Number of samples with tomato spotted wilt-like symptoms from mostly resistant (*Sw*-5b) processing tomato varieties from fields in the central (Fresno, Merced, San Joaquin, Stanislaus counties), northern (Colusa, Sutter and Yolo counties), and southern (Kern County) production areas in which the YPT and CPN strains of resistance-breaking (RB) tomato spotted wilt virus (TSWV) were detected by RT-PCR tests.

Year	County	Fields^a^	samples^b^	CPT^c^	YPT^c^	CPN^c^	Mixed^d^
2017	Contra Costa	---	2	0	2	---	---
	Merced	---	9	0	9	---	---
	Fresno	---	94	3	91	---	---
2018	Colusa	---	3^e^	0	0	---	---
	Yolo	---	20^e^	0	0	---	---
	Merced	---	41^e^	0	16	---	---
	Fresno	---	119^e^	0	97	---	---
	Kern	---	19^e^	0	14	---	---
2019	Colusa	---	4^e^	0	0	---	---
	Fresno	---	108^e^	1	95	---	---
2020	Yolo/Colusa	9	54^e^	0	0	---	---
	San Joaquin	4	16^e^	0	13	---	---
	Merced	3	27^e^	0	17	---	---
	Fresno	31	83^e^	0	64	---	---
	Kern/Kings	2	7^e^	0	0	---	---
2021	Colusa	1	1	0	1	---	---
	Yolo	6	39	5	34	---	---
	San Joaquin	3	33	8	25	---	---
	Merced	1	4	0	4	---	---
	Fresno	4	61	0	61	---	---
2022	Colusa	1	2	0	2	---	---
	Sutter	2	7	0	7	---	---
	Yolo	10	36	0	36	---	---
	San Joaquin	2	5	0	5	---	---
	Stanislaus	1	1	0	1	---	---
	Merced	2	6	0	6	---	---
	Fresno	9	29	0	29	---	---
2023	Colusa	6	49	0	10	37	2
	Sutter	2	12	0	4	8	0
	Yolo	10	141	0	63	75	3
	San Joaquin	2	6	0	6	0	0
	Fresno	11	43	0	43	0	0

^a^ Indicates number of fields from which samples were collected data not available for 2017–2019.

^b^ Samples were selected based on presence of spotted wilt-like symptoms, but not all were infected with TSWV.

^c^CPN and YPT RB strains were detected with specific RT-PCR tests, whereas CPT non-RB isolates were identified based on a combination of RT-PCR (with wild-type and general NSm primer pairs), sequencing NSm fragments, results of immunostrip tests to confirm TSWV infection, and failure to detect the *Sw*-5b gene.

^d^Mixed infection = sample was positive for infection with CPN and YPT RB TSWV strains;—no results because RT-PCR test not yet developed.

^e^For these samples the large number of negative results reflects a combination of having the symptoms caused by other factors (e.g., early curly top symptoms; nutrient deficiency, especially phosphorous; and even above ground symptoms of *Fusarium* spp. infections. Additionally, there was evidence of genetic diversity in non-RB strains in the northern production area because of the failure to detect non-RB strains with the WT primer pair designed based on sequences of an isolate from Fresno County.

Processing and fresh market tomatoes are important crops in California, and represent 95% and 30% of U.S. production, respectively, and 30–35% of world production of processing tomatoes. The Mediterranean climate of California (cool wet winters and hot dry summers) is favorable for this warm weather crop. Processing tomatoes tolerate higher temperatures (77–95 F or higher with adequate moisture) and are grown in the Central Valley from late January to mid-October. In contrast, fresh market tomatoes require lower temperatures (70–80 F) and have been grown on poles in open fields in coastal counties of Southern California, e.g., San Diego County. Both types of production involve the planting hybrid varieties transplants and using drip irrigation; whereas processing tomatoes are harvested mechanically and fresh market by hand as green or vine-ripe fruits.

To rapidly and specifically detect RB TSWV strains in large numbers of samples, we developed and employed RB strain-specific RT-PCR tests. Specific primers were designed based on alignments of NSm sequences of RB and non-RB strains, identifying nt changes (point mutations) associated with RB, and incorporating these into the 3’ end of a RB strain-specific forward primer. When a RB strain specific primer is paired with the general reverse NSm primer, a target ~440bp NSm fragment is amplified from total RNA (tRNA) of samples infected with that RB strain. TRNA was extracted from tomato samples with RNeasy Kit as per the instructions of the manufacturer (QIAGEN, Hilden, Germany). For cDNA production, 1–3 μg of RNA was used for reverse transcriptase reaction with virus-specific reverse primer of the NSm gene or random primer in the case of C118Y, T120N and WT TSWV strains detection, and the RevertAid kit (Thermo Fisher Scientific, Waltham, MA). Primer pairs were validated in RT-PCR tests with tRNA of tomato plants infected with the target RB strain, other RB strains, and a non-RB strain (non-RB) TSWV isolate; and of uninfected plants.

For spotted wilt samples from resistant varieties that tested negative for the C118Y RB strain and positive for the *Sw-5b* gene, we identified potential new RB strains by RT-PCR amplification of an ~1.2 kb NSm gene fragment with a general NSm primer pair (TSWV M1F AGAGCAATCAGTGCATCAGAA and TSWV M1200R GACCAAAGTTTGGCTTTTCAGC). RT-PCR-amplified fragments were directly sequenced with the TSWV M1F and TSWV M1200R primers and sequences analyzed for mutation in the ~20 aa region with the Sw-5b recognition site [19]. The presence of the *Sw-5b* gene was determined by a PCR test with a primer pair used in marker-assisted selection of this gene: *Sw-*5-2F and *Sw-*5-2R, which direct the amplification of an ~0.6 kb DNA fragment [26, 27]. The non-RB isolates were identified based on a combination of RT-PCR (with wild-type and general NSm primer pairs), sequencing NSm fragments, results of immunostrip tests to confirm TSWV infection, and failure to detect the *Sw-5b* gene.

The pathogenicity of selected isolates was determined by mechanical inoculation of tomato plants (second true leaf stage) of susceptible (cv. Glamour) and resistant (cvs. N6415 and HM3888) varieties as previously described [19]. Briefly, this involved mechanical inoculation of leaves of nine plants per variety with each isolate and across three independent experiments (Table 2).

**Table 2:** Results of pathogenicity tests of selected isolates of resistance-breaking (RB) and non-RB strains of tomato spotted wilt virus (TSWV) from spotted wilt samples collected from fresh market and processing tomato production in California following mechanical inoculation of resistant (Sw-5b) and susceptible tomato varieties.

			*Sw*-5b (+)		*Sw*-5b (-)
Sample ID	State/County	Strain	N6415	HM3888	Glamour
CPN-23-26-CA-Y	CA, Yolo	CPN	9/9 (4)^a^	9/9 (3)	9/9 (3)
CPN-23-38-CA-C	CA, Colusa	CPN	9/9 (3)	9/9 (3)	9/9 (3)
CPN-23-49-CA-S	CA, Sutter	CPN	9/9 (3)	9/9 (3)	9/9 (3)
YPT-23-51-CA-S	CA, Sutter	YPT	9/9 (2)	9/9 (2)	9/9 (2)
FPT-21-538-CA-SD	CA, San Diego	FPT	9/9 (2)	9/9 (2)	9/9 (2)
CPT-21-548-CA-SD	CA, San Diego	CPT	0/9 (0)	0/9(0)	9/9 (4)
FPT-23-24-CA-SD	CA, San Diego	FPT	9/9 (2)	9/9 (2)	8/9 (2)
YPT-22-730-NJ	NJ, Cedarville	YPT	9/9 (2)	9/9 (2)	9/9 (2)

^a^Number of plants infected/total plants inoculated and mean disease rating for 9 plants/isolate (total of three independent experiments) shown in parenthesis. The rating scale is (0) no symptoms; (1) mild mosaic; (2) moderate symptoms of mosaic, yellowing, and necrosis of leaves; (3) severe symptoms of leaf deformation, yellowing and necrosis; and (4) very severe symptoms including stunting and yellowing and necrotic spots of leaves, petioles and stems.

To investigate evolutionary relationships, complete NSm nt sequences were determined for selected RB and non-RB isolates collected in the present study (S1 Table) and, together with sequences available from GenBank, a dataset was generated. Codon-based alignment was performed with TranslatorX v1.0 [28], and Gblocks [29] (http://phylogeny.lirmm.fr/phylo_cgi/) was applied to eliminate poorly aligned positions without allowing for gap positions. Bayesian Inference (BI) and maximum likelihood (ML) phylogenetic trees were inferred using MrBayes v3.2.7 [30] and PhyML v3.1 [31], respectively. For BI the general time reversible (GTR) with gamma distribution (Γ4), an invariant (I) sites model was used with two runs of 5 million Markov chain Monte Carlo (MCMC) each saving one tree every 1,000 MCMC, and the posterior probability (PP) as branch support of the consensus tree was summarized after a 25% burn-in, and convergence was judged with the average standard deviation of split (ASDS < 0.01). For ML, the model HKY85 was defined using SMS [32] within the PhyMl server (http://www.atgc-montpellier.fr/phyml/) and branch support was determined by the aLRT SH-like method [31].

## Results and discussion

### Development and validation of an RT-PCR test for the C118Y strain that emerged in Fresno, California, USA in 2016

**Fig 1:**
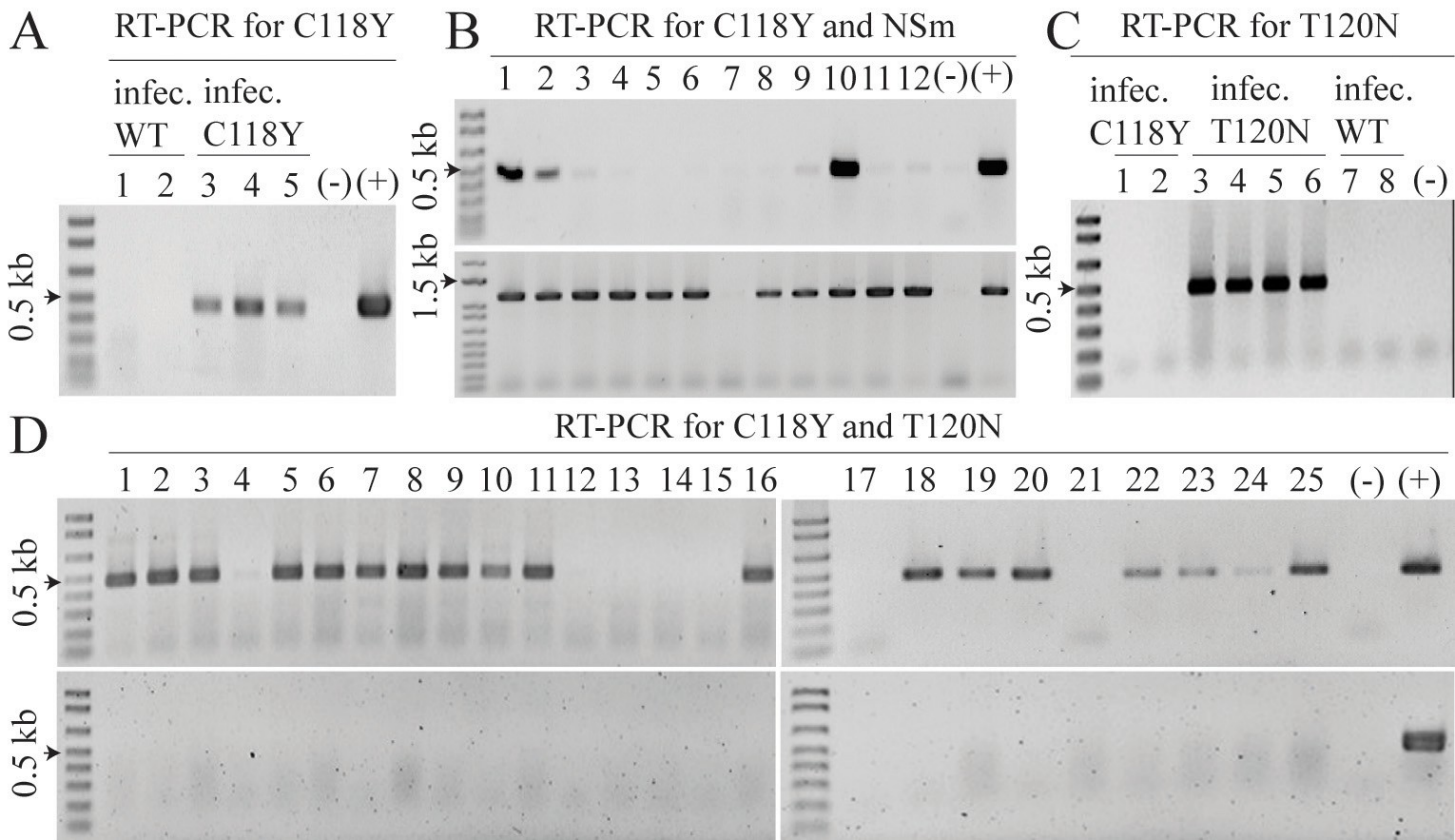
Development, validation and application of specific RT-PCR tests for rapid detection of C118Y and T120N resistance-breaking (RB) TSWV strains in spotted wilt samples from resistant (Sw-5b) varieties **A**, Validation of the specific RT-PCR test for the RB TSWV C118Y strain with the primer pair NSm-C118Y-338F and NSm-C118Y-799R and total RNA (tRNA) of: lanes 1 and 2, non-RB isolates of TSWV; lanes 3–5, isolates of the C118Y RB TSWV strain; and (-), tRNA of uninfected tomato plant and (+), tRNA of a known C118Y-infected tomato sample. **B**, Results of C118Y and NSm (TSWV- M1F and TSWV M1200R) RT-PCR test performed with tRNA of 12 spotted wilt samples from resistant varieties collected in Sutter County in 2023, with (-) and (+) as described in (A). **C**, Validation of the specific RT-PCR test for the RB TSWV T120N strain with the primer pair NSm-T120N-342F and NSm-C118Y-799R and tRNA of: lanes 1 and 2, isolates of the C118Y strain; lanes 3–6, isolates of T120N strain; lanes 7–8, isolates of non-RB TSWV; with (-) as described in (A). **D**, Results of C118Y and T120N RT-PCR tests of 25 spotted wilt samples from resistant varieties collected in Fresno County July 28, 2023, with (-) and (+) as described in (A).

Inspection of the alignment of NSm nt sequences from RB and non-RB isolates collected in 2016 from fields in the central production area revealed two nt changes at position 351 (A→C) and 353 and (G→A), respectively, generating the CTA triplet that codes for a Y residue at position 118 of the NSm protein. We designed the forward primer NSm-C118Y-338F-5’-CCGGCCAGGTCATCAAAAT**C**T**A**-3’, incorporating these nt changes into the 3’ end, and the six non-TSWV nts into the 5’ end to enhance specificity. When paired with the general reverse primer NSm-C118Y-799R-5’-GCTTCTCACTGTTTCCTTTAGG-3’ that anneals to a conserved sequence in the NSm gene and with the following RT-PCR conditions: 94°C for 5’; 25 cycles of (94°C for 15”, 62°C for 15”, 72°C for 30”) followed by 72°C for 5’, the target 460 bp NSm fragment was amplified from tRNA of plants infected with C118Y strain (Fig 1A). PCR reaction was performed in a SimpliAmp thermal cycler (Applied Biosystems, Waltham, MA). Each 25 μl PCR consisted of 16.4 μl water; 2.5 μl 10X PCR buffer; 1 μl of 10 μM primers (10 uM); 1 μl of 25 mM MgCl2; 1 μl of 10 mM dNTP; 3 μL of cDNA and 0.1 μL of 5U/μl DNA Taq polymerase (Apex, Genesee Scientific, Morrisville, NC).

In validation RT-PCR tests, the target 460 nt fragment was amplified from tRNA of tomato leaves infected with the C118Y RB strain, whereas no fragment was amplified from tRNA of plants infected with the non-RB isolate or from tRNA of uninfected plants (Fig 1A). This RT-PCR test was then used to detect the C118Y strain in all spotted wilt samples collected or received during this study (2017–2023). These samples also were tested for the *Sw-5b* gene, and all were confirmed to possess this gene based on the amplification of the 0.6 kb target fragment in the Sw-5b PCR test. This fragment was not amplified from DNA of non-Sw-5b (susceptible) varieties.

**Fig 2:**
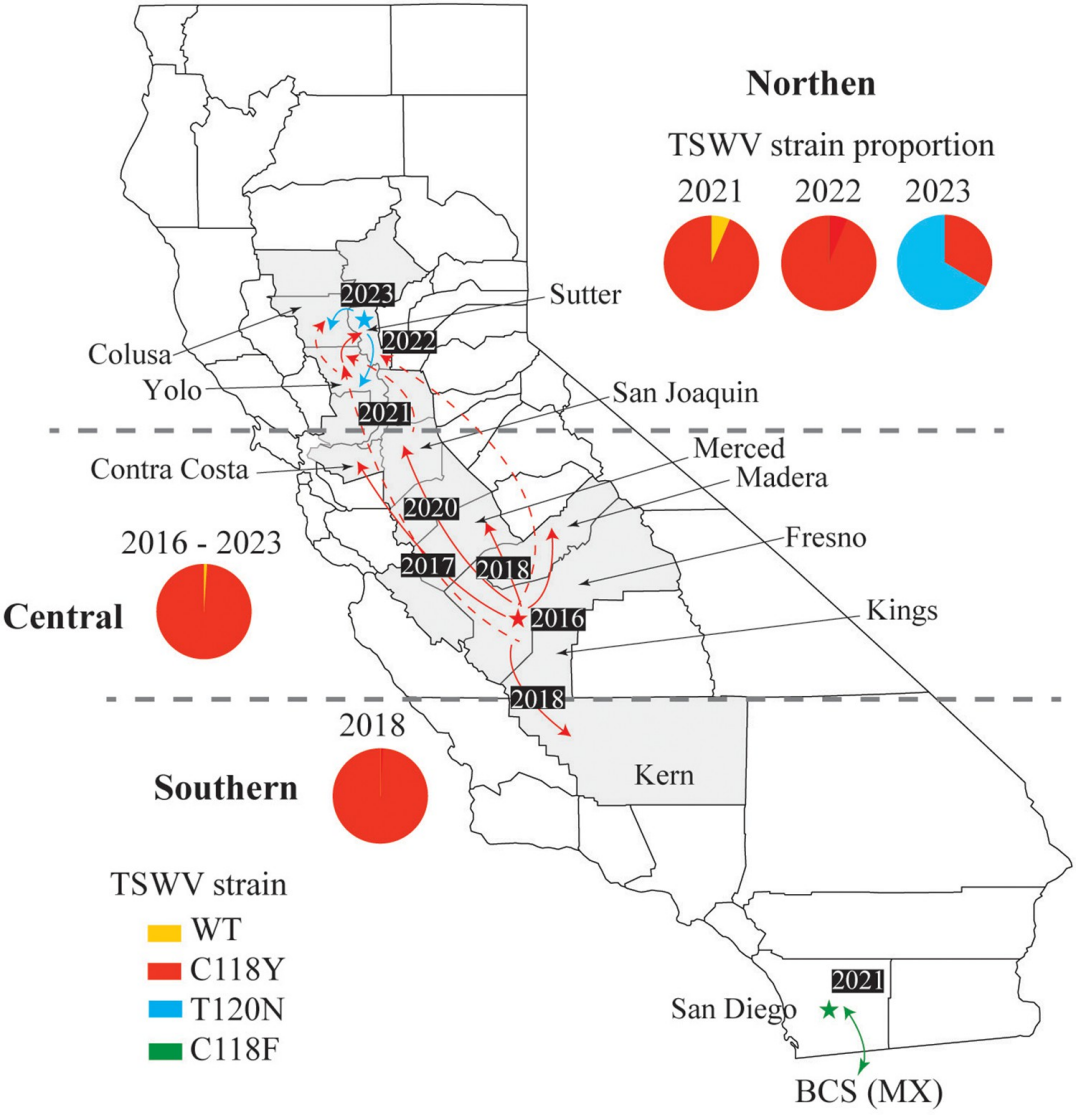
Map of the state of California (US) showing the emergence of resistance-breaking (RB strains of tomato spotted wilt virus (TSWV) in southern, central and northern processing tomato production areas (counties shown in gray) and fresh market areas (San Diego County and the state of Baja California de Sul [BCS] in Mexico [MX]). Colored stars and associated years show the first detections of the four RB TSWV strains in California, pie charts present proportion of RB and wild-type (WT) strains detected in processing tomato samples and years, whereas solid red arrows show spread to adjacent counties and dashed lines show potential long-distance spread and associated years.

In presenting the results of surveys and testing of spotted wilt samples from processing tomato fields in California from 2017–2023, we divide production into three areas northern (Colusa, Glenn, Sutter and Yolo counties), central (Fresno, Kings, Merced, Contra Costa, San Joaquin and Stanislaus counties) and southern (Kern County) (Fig 2). We also will present these results in respect to (i) before detection of RB strains in the northern production area (2017–2020), (ii) introduction and spread of the C118Y strain in the north (2021–2022) and (iii) emergence of the T120N strain in this area in 2023.

### Survey results for 2017–2020: C118Y strain rapidly becomes predominant in the central production area

From 2017 to 2020, the majority of samples tested were from the central production area (499/606 [82%]), and mostly from Fresno (404/499 [80%]) and Merced (77/499 [15%]) counties (Table 1). All of these samples were from fields planted with resistant (Sw-5b) varieties. In 2017, the year following the detection of the C118Y RB TSWV strain in Fresno [19], it was predominant in samples from the central production area (102/105 [97%]), whereas the non-RB strain was detected in only 3 samples (3%). This showed the persistence and rapid spread of this RB strain and is consistent with the widespread planting of varieties with the *Sw-5b* gene in this area (Fig 2). Similar results were obtained for samples from the central production area in 2018 (113/113 [100%]), 2019 (95/96 [99%]), and 2020 (94/94 [100%]). This RB strain was also detected in samples from the southern area in 2018 (14/14 [100%]), but not in 2020 (Table 1). Here it should be noted that most of the spotted wilt-like samples that tested negative for the C118Y strain had symptoms caused by other factors, e.g., foliar symptoms of *Fusarium* spp. or were in poor condition.

In contrast, spotted wilt in the northern production area during this period (2017–2020) was not economically important, and the planting of resistant varieties provided effective management of the non-RB isolates in the area. Furthermore, relatively few spotted wilt samples were tested in 2017–2019, whereas the number of samples increased in 2020, but these were from susceptible varieties infected with non-RB isolates. Notably, many of these non-RB isolates were not detected with the RT-PCR test for the non-RB isolate from Fresno (Table 1), or were caused by other factors as previously noted. Together, these results revealed an overall lower incidence of spotted wilt in the northern production area during this period, and also suggested that genetic diversity exists among non-RB strains in the central and northern production areas.

Thus, the surveys conducted from 2017 and 2020 revealed the predominance and spread of the C118Y strain in the central production area (Fresno, Merced, Contra Costa and San Joaquin counties) (Fig 2). This strain was detected in samples from the southern area (Kern County) in only 2018, suggesting it has not become established, which is consistent with spotted wilt being less economically important in this area. The failure to detect the C118Y strain in samples from the northern production area during this period suggested this RB strain had not spread to this production area. This finding was in agreement with the lower spotted wilt incidence in processing tomato fields in this area, and some planting of non-resistant varieties.

### Survey results for 2021 and 2022: Spread of the C118Y strain to the northern production area

The RB TSWV situation changed in 2021 and 2022 because the C118Y strain was detected in spotted wilt samples from resistant varieties in fields in the northern production area. Thus, with the planting of more resistant varieties in this area, we began we began to receive more spotted wilt samples to receive more spotted wilt samples from field planted with these varieties and nearly all were infected with the C118Y strain (Table 1). Most of the detections were for samples from Yolo County, but also in samples from Colusa and Sutter counties (Table 1). Thus, in 2021 and 2022, 35/40 (88%) and 45/45 (100%) of spotted wilt samples were positive for infection with the C118Y RB strain, whereas non-RB isolates were only detected in 5 samples (12%) in 2021. These results showed that ~5 years following the emergence of the C118Y strain in Fresno County, it had spread to the northern production area in 2021 and became established in 2022 (Fig 2).

As expected, samples of resistant varieties with spotted wilt symptoms from the central production area in 2021 and 2022 were mostly infected with the C118Y strain, i.e., 90/98 (92%) and 40/40 (100%), respectively. These results are fully consistent with the predominance of this strain in this area (Fig 2), where it emerged in 2016. This reaffirmed the presence of the C118Y strain in counties in which it was previously reported, as well as demonstrating the spread and persistence in the northern production area. Moreover, given that i) the C118Y strain is aggressive and virulent, ii) RB strains increase fecundity of the thrips vector and iii) that resistant varieties are now widely planted in the northern production area, spotted wilt outbreaks are likely to become more common, potentially economically important and require management efforts.

### 2023 survey results: Emergence of a New RB TSWV strain in the northern production area

In 2023, as part of a project to investigate unusual outbreaks of curly top disease (CTD) caused by beet curly top virus (BCTV) in the northern production area, we monitored processing tomato fields in Colusa and Yolo counties for CTD beginning in early May and placed yellow sticky cards (YSCs) around these fields to detect migrating of beet leafhoppers [33]. Resistant (Sw-5b) processing tomato varieties were planted in these monitored fields, as well as in most other fields in the northern production area in 2023. The spring weather conditions in 2023 were cool and wet, and processing tomato fields were planted later than usual, i.e., in late April-early May rather than late March-early April [33]. Furthermore, virtually no CTD was observed in our monitored fields nor in other processing tomato fields in the northern production area and few beet leafhoppers were captured on YSCs. This dramatic decrease in CTD incidence in processing tomatoes in the northern production area was attributed to the cool wet spring weather conditions [33].

**Fig 3:**
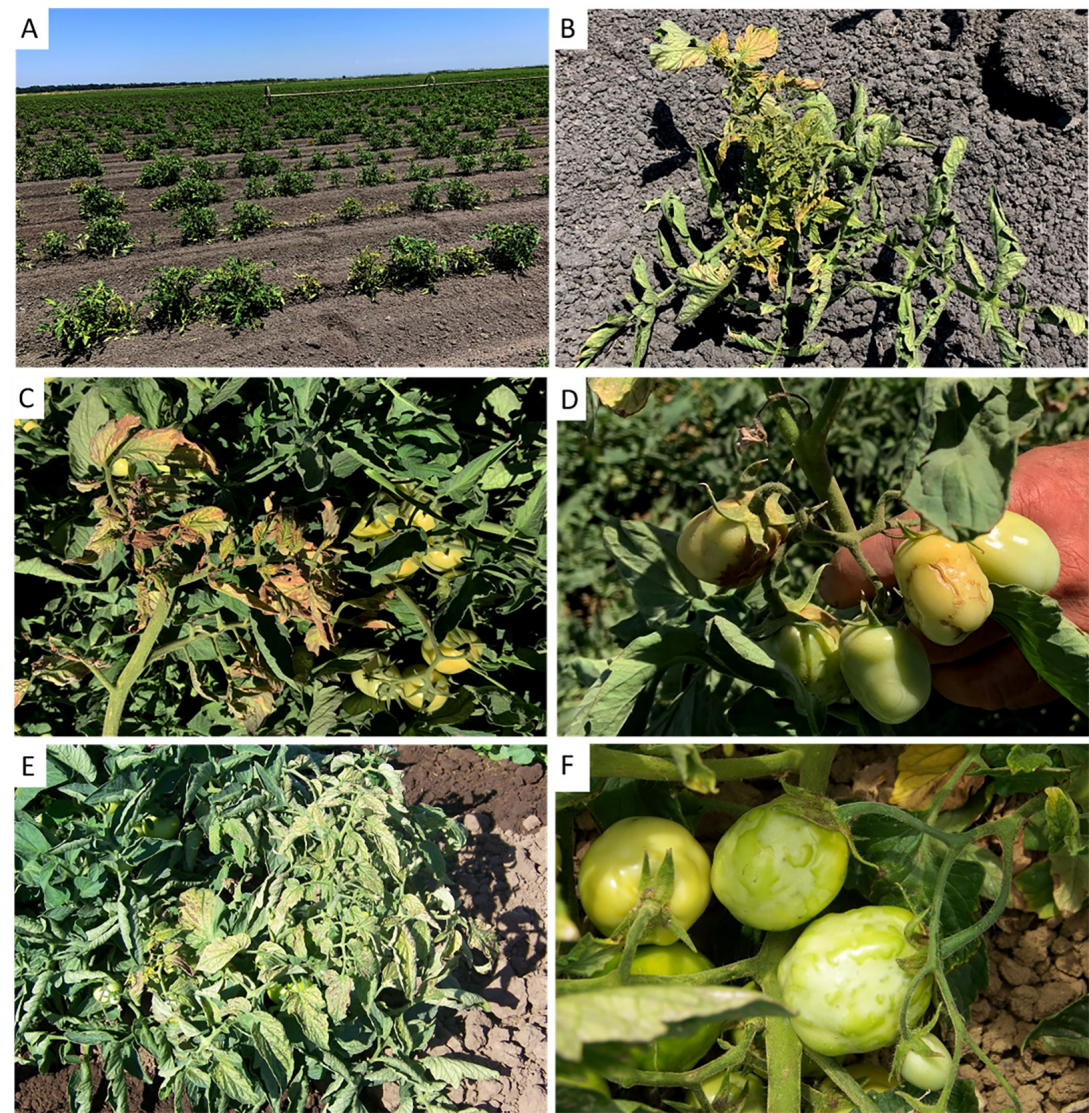
An unusual spotted wilt symptom phenotype observed in tomato plants of resistant processing tomato varieties infected with resistance-breaking (RB) strains of tomato spotted wilt virus (TSWV) in the central (C118Y strain) and northern (C118Y and T120N strains) production areas in 2023. **A**, Late-planted field in Colusa County with a high incidence (>50%) of infection with RB TSWV and with plants showing severe stunting and striking chlorosis of leaves. **B**, Close-up of a plant from the field in (A) showing the severe chlorosis, curling and purpling of leaves and the onset of scorching (necrosis). **C**, Close-up of the severe chlorosis, curling and scorching (necrosis) of leaves and relatively mild fruit symptoms. **D**, Necrotic ring on a green fruit (rarely observed). E, More typical symptoms of TSWV infection of a tomato plant, showing chlorosis and necrotic spots on leaves and F, Bumpiness, chlorotic ring spots and deformation of tomato fruits.

During our surveys of these fields, we observed low incidences (<1%) of spotted wilt symptoms beginning in late May-early June. Furthermore, although few beet leafhoppers were captured on YSCs placed around these fields, adult thrips were regularly captured. During this time, Farm Advisors, PCAs and growers were reporting spotted wilt symptoms in resistant varieties in many commercial fields in the northern production area, though mostly at low incidences (<5%). By July-August, the spotted wilt incidence in our monitored fields and in most other fields remained relatively low (<5%). This may relate to low levels of primary inoculum and thrips management efforts, i.e. application of insecticide. However, in some ‘hotspot’ areas of Colusa and Yolo counties, fields with higher incidences (~25% or greater in some areas of fields) were reported. In addition, growers and PCAs were reporting unusually severe foliar spotted wilt symptoms, striking yellowing, purpling and scorching (Fig 3A).

Surveys in Colusa County in early July revealed some late-planted fields with early infection and high incidences of spotted wilt symptoms (~50% in some parts of fields) (Fig 3A). Furthermore, it was noted that the spotted wilt symptoms in tomato plants in these and other fields had developed the unusual symptoms characterized by strong yellowing, purpling and scorching of leaves; ringspots on stems; and relatively mild fruit symptoms, mostly bumpiness and occasional necrotic rings (Fig 3A–3D, compare to typical symptoms observed on leaves and fruits Fig 3E and 3F). Thus, these results showed increased spotted wilt pressure in the northern production area in 2023, as well as an unusual spotted wilt symptom phenotype in some fields. Indeed, the spotted wilt outbreaks in resistant varieties in the northern production area in 2023 resulted in an increased number of samples received for testing for RB TSWV infection (Table 1).

We no longer receive large numbers of spotted wilt samples from resistant varieties in the central production area as growers and PCAs are aware of RB TSWV, However, 201 spotted wilt samples were tested from this area in 2023, compared with 45, 40, 54, 4, 23 and 0 samples tested for the years 2022–2017 (Table 1).

We now received fewer spotted wilt samples for testing from the central production area because RB TSWV has been present for seven years (Table 1). However, PCAs, growers and industry personnel in the central production area were also reporting the unusual spotted wilt symptom phenotype in some fields. In 2023, the 49 spotted wilt samples from the central production area were from Fresno (43/49 [88%]) and San Joaquin (6/49 [12%]) counties. Many of the Fresno samples were collected during a field visit in late July in which we surveyed for virus symptoms and collected spotted wilt samples from an experimental field trial and four commercial processing tomato fields. Spotted wilt symptoms were observed in all fields and at low incidence (<5%) in the experimental trial and two commercial fields in which the disease came in late. Two fields had higher incidences of ~15–20%, including one early-planted organic field near the foothills. Importantly, processing tomato plants in all of these fields showed the unusual spotted wilt symptom phenotype, observed in fields in the northern production area. This showed that these unusual spotted wilt symptoms were not specific to the northern production area.

In 2023, we initially tested spotted wilt samples from fields of resistant varieties in the northern production area for infection with the C118Y strain, which was prevalent in this area in 2022 (Table 1). This was also based on our experience with this strain in the central production area. Therefore, we hypothesized that the C118Y strain would be predominant in 2023 spotted wilt samples from the northern production area. Initially, samples from our monitored fields in Yolo County were infected with the C118Y strain. However, as more spotted wilt samples were received from fields in Colusa, Sutter and Yolo counties in June and July, many were unexpectedly negative for infection with the C118Y strain. This was especially the case for samples from Colusa and Sutter counties (Table 1). Moreover, because these samples showed obvious symptoms of spotted wilt, and were positive for TSWV infection based on results of immunostrip tests (for all that were tested) and for the *Sw*-*5b* gene. This situation is shown in Fig 1B, in which the C118Y strain was detected in 3 of 12 spotted wilt samples from resistant varieties in a field in Sutter County in 2023. With the exception of one sample that did not have spotted wilt symptoms, the other eight samples had typical spotted wilt symptoms and were positive for TSWV infection based on immunostrip test results and for the *Sw*-*5b* gene. These results raised the possibility that these samples were infected with a different RB TSWV strain.

To further investigate the possibility that a new RB strain was infecting these eight samples that tested negative for C118Y infection, we performed a second RT-PCR test with the general NSm primer pair (TSWV M1F and TSWV M1200R) and tRNA of these samples. As shown in Fig 1B, the expected-size ~1.2 kb NSm fragment was amplified from all eight samples, confirming infection with TSWV. Importantly, this fragment includes the sequence encoding the ~20 aa Sw-5b recognition region of the NSm [12]. These fragments were sequenced, and an alignment generated with these and other NSm sequences as described. Inspection of this alignment revealed that the TSWV isolates infecting these eight samples from Sutter County all had a C→A nt change at position 359 of the NSm gene, which generates the triplet AAT that encodes for an acidic asparagine residue (N) at aa position 120, instead of the threonine (T) residue in the non-RB TSWV isolates. Thus, these results indicated that these eight samples were infected with a different RB strain, T120N, thereby explaining the negative results obtained with the RT-PCR test for the C118Y strain. The T120N RB strain has been reported from Spain, but not from the NW [18]. Thus, spotted wilt symptoms in resistant varieties in the northern production area in 2023 were caused by two RB TSWV strains: C118Y and T120N. The detection of the T120N strain was detected in most samples from Colusa and Sutter counties, suggested it may have originated in this area.

Because the new T120N RB strain was infecting many of the spotted wilt samples from the northern production area in 2023, we next developed a specific RT-PCR test for the detection and monitoring of this new RB strain. We used an alignment of NSm sequences of isolates of C118Y and T120N RB strains and non-RB TSWV isolates from the northern production area. Inspection of this alignment revealed the C→A nt change at position 359 was the only nt change in this region of the NSm gene. Thus, to develop a specific RT-PCR test for the T120N strain, the nt A was incorporated into the 3’ end of a forward primer and the 6 non-TSWV nts into the 5- end as previously described to generate the primer: NSm-T120N-342F-5’-CCGGCCAGGTCATCAAAATCTGTCCAA**A**-3’. This primer was then paired with the general NSm-C118Y-799R reverse primer (used in the C118Y RT-PCR test) and used in RT-PCR tests with tRNA of spotted wilt samples infected with the T120N strain and from uninfected plants. Results of these tests revealed that the expected-size 440 bp fragment was amplified only from tRNA of samples infected with the T120N strain (Fig 1C). RT-PCR conditions: 94°C for 5’, 25 cycles of (94°C for 15”, 64.5°C for 15”, 72°C for 30”) and 72°C for 5’. For validation, we used tRNA from spotted wilt samples infected with i) the C118Y strain, ii) T120N strain, iii) non-RB strain and iv) uninfected tomato leave tissue. In these tests, the expected-size 440 bp NSm fragment was amplified only from tRNA of samples infected with the T120N strain (Fig 1C), whereas no fragment was amplified from tRNA of plants infected with the C118Y RB strain, the non-RB strain or from tRNA of uninfected control plants (Fig 1C). In 2023, all spotted wilt samples received or collected from the northern production area were tested for infection with the T120N strain specific RT-PCR test to monitor for this new strain.

Results of testing of 2023 spotted wilt samples from the northern and central areas with the RT-PCR tests for the C118Y and T120N strains are presented in Table 1. Samples from Colusa and Sutter counties had higher rates of infection with the T120N strain (47/61 [77%]) compared with the C118Y strain (14/61 [23%]), and this was for samples collected early (May-June, 38/52 [75%]) and late (July-August, 6/8 [75%]). The infection rates for samples from Yolo County were more similar for these RB strains, 53% (75/141) T120N compared with 45% (63/141) C118Y and 2% mixed infection (3/141).Early collected samples (May to June) from Yolo County again had higher rates of C118Y infection (39/63 [62%]) compared with T120N (24/63 [38%]), whereas later samples (July to August) had higher rates of T120N infection (51/75 [68%]) compared with C118Y (24/75 [32%]). These results revealed the rapid spread of the T120N strain into Yolo County, most likely from Colusa or Sutter County via winged viruliferous adults of the *F*. *occidentalis* supervector, which is prevalent in the northern production area [5]. These results also indicated that the T120N strain effectively competed with the C118Y strain in Yolo County, based on the increased number of detections in samples collected later in the growing season. It is also interesting that although the C118Y and T120N strains overlapped in Yolo County in 2023, there were relatively few mixed infections detected (Table 1). This may indicate some level of interference between these strains [34], making something interesting to investigate further. Thus, the T120N strain emerged and spread rapidly in the northern production area in 2023 and, remarkably, was the most commonly detected strain by the end of the growing season (Table 1).

However, we did not find evidence of an association of infection with the ‘new’ T120N strain and the unusual spotted wilt symptom phenotype. First, samples from tomato plants with these symptoms were infected with T120N or C118Y strains and rarely with both. Second, these symptoms were observed in fields in the northern and central production areas and we found no evidence that the T120N strain had spread to the central production area (see below).

Finally, results of RT-PCR tests of the 43 spotted wilt samples from Fresno County, including many from plants with the unusual symptom phenotype, showed that all were infected with the C118Y strain (Table 1 and Fig 1D), whereas the T120N strain was not detected in any of these samples (Table 1 and Fig 1D). These results (i) were consistent with the predominance of the C118Y strain in the central production area, and (ii) suggested that the T120N strain had not spread to this area. We suspect that this symptom phenotype may reflect the genetics of certain hybrid varieties, once the Sw-5b resistance has been overcome.

### Survey results: Fresh market tomato production

We also received samples from fresh market tomato production from multiple locations. The first ‘samples’ were tRNA extracts from spotted wilt samples collected from resistant varieties being cultivated in protected culture (screenhouses) in the state of Baja de Sul, Mexico in 2014. Using RT-PCR and the general NSm primer pair, the target NSm fragment was amplified and sequenced as previously described. Based on these results, the C118Y RB TSWV strain was detected in tRNA of three samples from one location, whereas the C118F strain was detected in tRNA of two samples from another location. These results showed that the spotted wilt outbreaks in resistant varieties in Baja, Mexico in 2014 were caused by at least two RB TSWV strains: C118Y and C118F. Importantly, this represents one of the first detections of the C118Y RB strain in the NW, whereas the C118F motif had not been associated with RB in 2014. Recently, the C118F strain was reported associated with spotted wilt in resistant fresh market tomato production in Mexico, and isolates were confirmed to infect and cause spotted wilt symptoms in Sw-5b varieties [22]. Notably, as part of this study, we also performed the same analysis of five non-RB (control) samples collected from non-Sw-5b processing tomato varieties in Fresno in 2014 (pre-C118Y). All of these had the CPT motif associated with non-RB strains.

In 2021, we received 11 samples from open-field fresh market tomato production in San Diego County, CA. The foliar symptoms were mostly chlorosis and necrotic lesions, whereas fruits were bumpy and had light-colored blotches. TSWV infection in these samples was demonstrated based on positive results with TSWV immunostrip tests, and these samples also were positive for the *Sw*-*5b* gene. However, all these samples were negative for infection with the C118Y RB TSWV strain based on results of the specific RT-PCR test. However, amplification and sequencing of the 1.2 kb NSm fragments revealed that 3 samples were infected with the C118F RB TSWV strain. Interestingly, samples received from this location in 2023 showed more typical spotted wilt symptoms and were also infected with the C118F strains. In October 2022, we received 7 samples of spotted wilt from a resistant fresh market variety being grown in New Jersey, US and all were positive for infection with the C118Y strain.

**Fig 4:**
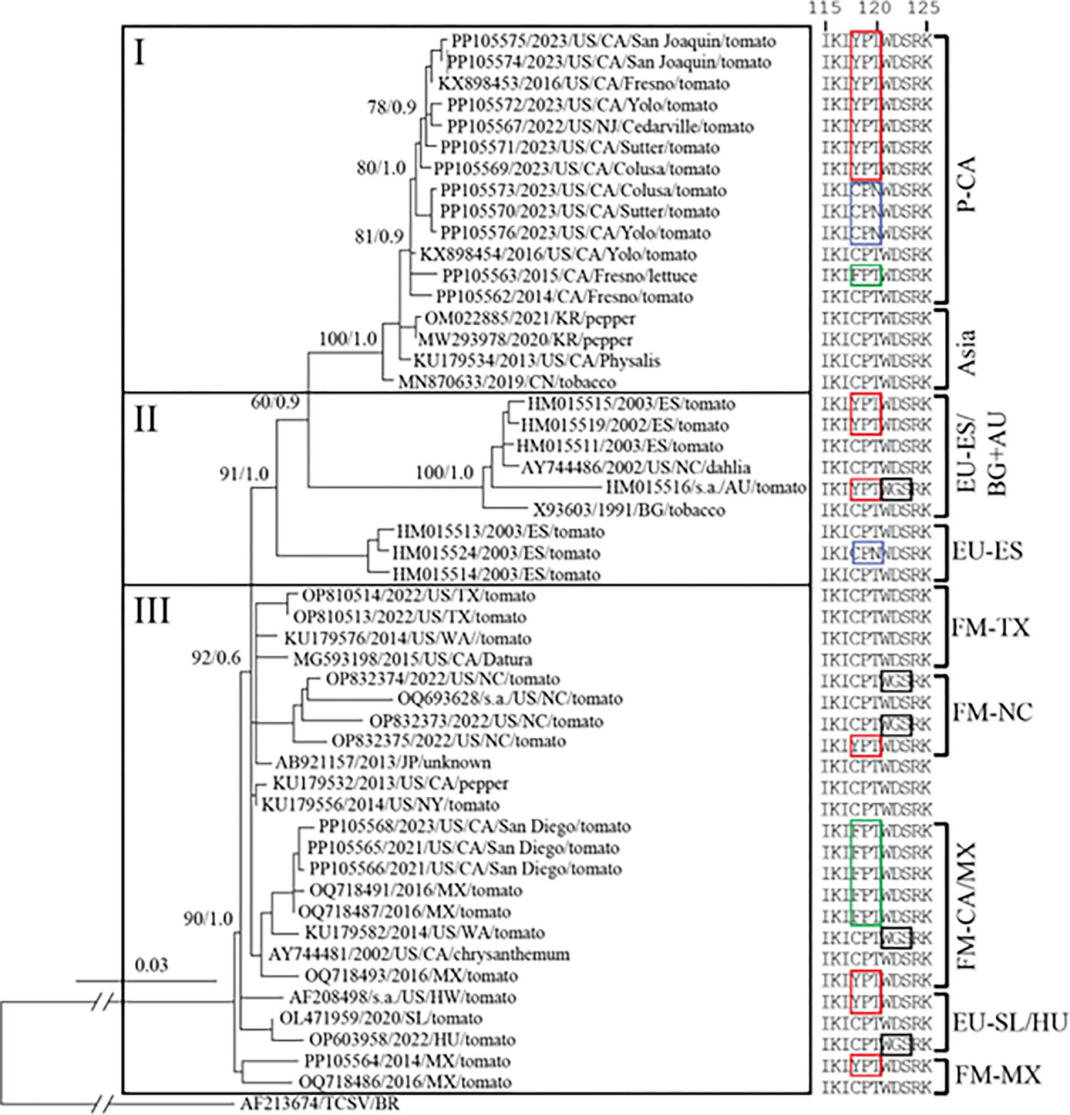
Phylogenetic tree constructed with the complete nucleotide sequences of NSm genes of isolates of tomato spotted wilt virus (TSWV) that overcome resistance conferred by the *Sw*-*5b* gene (resistance-breaking, RB) or do not have this capacity (non-RB). The dataset used in the alignment includes a total of 51 sequences, including 15 from fresh market and processing tomato samples determined in the present study and 36 retrieved from GenBank. The tree was rooted with the complete NSm sequence of an isolate of tomato chlorotic spot virus (TCSV) (outgroup). An eleven amino acids (aa) sequence of the NSm movement protein that includes the recognition site for Sw-5b, and the positions of aa changes associated with RB TSWV strains is shown in colored boxes [color key: red = C118Y, blue = T120N, green = C118F, and black = D122G]. Relevant approximate likelihood ratio test (for maximum likelihood) and posterior probability (for Bayesian analysis) values are shown on branches. Subclades are labeled as follows: P-CA: processing tomato-California; EU-ESP/BG+AU: Europe-Spain, Bulgaria and Australia; EU-ESP: Europe-Spain; FM-TX: fresh market tomato-Texas; FM-NC: fresh market tomato-North Caroline; FM-CA/MX: fresh market tomato-California and Mexico; EU-SL/HU: Europe-Slovenia and Hungary; and FM-MX: fresh market tomato-Mexico.

Finally, in 2019, we received five spotted wilt samples from a non-resistant variety in Ventura County. These samples also tested negative for infection with the C118Y strain, and were probably infected with non-RB TSWV based on symptoms, positive immunostrip tests and negative for the *Sw-5b* gene. A map of the states of California, U.S.A., showing the emergence and spread of resistant breaking strains tomato spotted wilt virus (RB-TSWV) in processing tomato production areas are shown in Fig 4.

### Pathogenicity testing

In our previous studies with the C118Y strain from Fresno we used mechanical inoculation of resistant and susceptible processing tomato varieties to establish that this strain overcame Sw-5b resistance and was more virulent than a non-RB strain in susceptible varieties [19]. Thus, we next used mechanical inoculation experiments with isolates of the T120N strain from Colusa (T120N-23-38-C), Sutter (T120N-23-49-S) and Yolo (T120N-23-26-Y) and showed all three induced severe spotted wilt symptoms of strong leaf chlorosis and necrosis (ratings of 3 for 0–4 scale) in all inoculated plants of the resistant and susceptible varieties (Table 2). The T120N-23-26Y isolate induced particularly severe symptoms in the N6415 variety (rating of 4). In equivalent inoculations, a non-RB CPT strain (CPT-21-548-CA-SD) induced very severe symptoms in the susceptible cv. Glamour, no symptoms in plants of the two resistant varieties whereas no symptoms developed in mock-inoculated plants (Table 2). Together, these results demonstrated that isolates of the ‘new’ T120N strain from California are highly virulent and overcome the *Sw*-*5b* resistance gene. This is fully consistent with the rapid emergence and spread of this strain in the northern production area in 2023.

In addition, we tested the pathogenicity of isolates of the C118Y strain from: (i) a processing tomato sample from Sutter County CA collected 2023 (C118Y-23-51-S) and the fresh market sample from New Jersey collected in 2022 (C118Y-22-730-NJ); and two isolates of the C118F strain from San Diego County collected in 2021 (C118F-21-538-CA-SD) and 2023 (C118F-23-24-SD) (Table 2). All four isolates infected all inoculated plants of the susceptible and resistant varieties, but the disease symptoms were less severe (ratings of 2) compare with those induced by isolates of the T120N strain (Table 2). Thus, these isolates induced a range of symptoms in infected plants, which varied depending on the isolate, and tomato variety (Table 2).

### Phylogenetic analysis

To gain insight into the evolutionary relationships among RB TSWV isolates/strains identified in California in the present study, an alignment of complete NSm nt sequences of (i) 8 isolates of RB TSWV C118Y and T120N strains from the northern and central processing tomato production areas in 2023; (ii) one non-RB isolate from Fresno collected in 2014 (before emergence of the C118Y RB strain); (iii) one C118F RB isolate from lettuce from Fresno County in 2015; (iv) three C118F RB isolates from fresh market tomato in San Diego County (2021 and 2023), (v) two C118Y RB isolates from fresh market tomato from New Jersey (2022) and Mexico (2014); (vii) 36 complete NSm sequences from GenBank, including the known RB-TSWV strains C118Y, T120N, D122G, and C118F; and vii) the NSm nt sequence of an isolate of tomato chlorotic spot virus (TCSV) as an out-group taxon. The dataset with these sequences was used to generate phylogenetic trees with BI and ML approaches. The trees showed similar topology, and the tree generated with the BI approach is presented in Fig 3.

As shown on the left side of the phylogenetic tree, three major clades or lineages were identified and named I-III. Lineage I includes a large subclade that includes all isolates from processing tomatoes in California. Included in this subclade were: i) the non-RB isolate from Fresno from 2014 (pre-C118Y); ii) the C118Y strain from Fresno collected in 2016; iii) isolates of the C118Y strains from the central and northern production areas; iv) isolates of the T120N strain from the northern production area; and v) the C118F strain from lettuce in Fresno collected in 2015 (Fig 3). A small subclade with isolates from Asia and one from the US was included in Lineage I (Fig 3). Lineage II includes isolates from Europe, mostly Spain, as well as isolates from Australia and the US. Importantly, lineage II includes isolates of RB strains C118Y, T120N and D122G (Fig 3). This lineage was divided into two well-supported subclades, one with isolates from Spain and other countries; whereas the other only had isolates from Spain, including the isolate of the T120N RB strain (Fig 3). The isolate from the US dates back to 2002 and is a non-RB isolate, whereas the Australian RB strain is unique in having the ‘C118Y - D122G’ motif, which involved two aa substitution mutations (Fig 3). Together, these results indicate i) that these RB TSWV strains are polyphyletic, and ii) the C118Y and T120N RB strains infecting processing tomatoes in California evolved locally from existing non-RB isolates rather than being introduced exotic strains (Fig 3). Consistent with this notion, it is worth noting that the C118F strain was detected in a lettuce sample with spotted wilt collected in Fresno County in 2015, and that this isolate was also placed in the large subclade with processing tomato isolates, rather than in the fresh market lineage with other FTP strains (Fig 3). The fact that isolates of this FTP strain were not encountered again in this study, even from subsequent lettuce samples with spotted wilt, suggests it may not have been competitive with the C118Y strain.

Lineage III includes RB and non-RB isolates from fresh market tomato production in Mexico and states of the US, mostly California, North Carolina and Texas (Fig 3). This clade shows the greatest genetic diversity and evolutionary history among isolates, including sequences of non-RB isolates collected >20 years ago (Fig 3). The subclades of lineage III generally reflect the geographic origin of isolates, e.g., subclades comprised of isolates from Texas, North Carolina, California/Mexico, Mexico, and even one with isolates from Europe (Fig 3). Furthermore, RB strains were placed in most of these subclades (Fig 3), consistent with local evolution and polyphyletic nature of these strains. For example, the CA/MX subclade includes isolates of the C118Y, C118F and D122G strains; whereas the North Carolina subclade has isolates of the C118Y and D122G strains [23, 25]. In contrast to the RB TSWV strains associated with processing tomatoes in California, the C118F strain was detected in samples from fresh market tomato production in Mexico and in states with associated fresh market production, e.g., California and Texas (Fig 3) [22].

Importantly, we confirmed the pathogenicity and RB capacity of an isolate of the C118F strain from Southern California (Table 2), in agreement with results for an isolate of C118F from Mexico [22]. The prevalence of the C118F strain in fresh market tomato varieties may reveal differences in the TSWV-host interaction and adaptation to the different genetics of fresh market and processing tomato varieties. The capacity of the TSWV genome, including the NSm gene, to rapidly adapt to host backgrounds has been recently demonstrated in passage experiments and HTS analyses [35]. However, for both types of tomato production in California, the widespread planting of resistant varieties has driven the local evolution of multiple RB strains (Figs 1 and 3).

The phylogenetic analyses also revealed evidence of spread over long distances. For example, the isolate of the C118Y strain from fresh market tomato production in New Jersey collected in 2022 was placed in the subclade with isolates from processing tomato production in California. In the FM-CA/MX subclade, the consistent clustering of isolates of the C118F strain from both countries suggests long distance movement in association with this production system.

Taken together, the results of the phylogenetic analyses further demonstrate the polyphyletic nature of RB TSWV strains, and how widespread planting of varieties with the strong, single dominant *Sw-5b* resistance gene has facilitated this process. This has occurred in processing and fresh market production, placing strong selection pressure on the virus to evolve RB strains [18]. Our results also revealed associations of some RB TSWV strains with fresh market or processing tomato production, suggesting different types of virus-host interactions. Indeed, an unusual spotted wilt symptom phenotype observed in 2023 was attributed to a general response of certain processing tomato hybrid genotypes to infection by RB strains in the absence of Sw-5b resistance.

## Conclusions

Our study documents the rapid emergence, spread and interactions of RB TSWV strains in processing and fresh market tomato production systems in California. This involved the use of RB strain-specific RT-PCR and pathogenicity testing and evolutionary analyses based on NSm sequences. We show that the C118Y strain first detected in Fresno in 2016 quickly became predominant in the central production area, spread to the northern production area in 2021 and became predominant in 2022. However, in 2023, the new T120N RB strain unexpectedly emerged in the northern production area. Monitoring with a specific RT-PCR test indicated this RB strain emerged in the area of Colusa and Sutter counties and spread into Yolo County, where it effectively competed with the existing C118Y strain. The rapid spread of new RB strains in these agroecosystems was mediated by large populations of the supervector *F*. *occidentalis* [5, 23] and widespread planting of new susceptible varieties with the single dominant *Sw*-*5b* resistance gene. Pathogenicity tests revealed confirmed that isolates of the California T120N strain were highly virulent on resistant and susceptible varieties. Phylogenetic analyses performed with complete NSm sequences indicated that RB strains in California emerged locally rather than being introduced exotic strains.

RB TSWV strains were identified in samples of fresh market varieties dating back to 2014, and an interesting association of the C118F RB strain was observed. The phylogenetic analysis also provided evidence of spread over long distances, e.g., introduction of the C118Y strain to the northern production area of California, and detection of isolates of the C118F strain in samples from Southern California and Mexico. In 2023, an unusual spotted wilt symptom phenotype was associated with the susceptible response of the underlying genetics of hybrid tomato varieties to infection with the C118Y or T120N strains of TSWV.

Finally, our results show the complex and rapidly changing nature of RB TSWV strains as the virus adapts to the thrips supervector and extensive planting of resistant varieties in fresh market and processing tomato production just in the California. Unfortunately, the effectiveness of the *Sw*-*5b* resistance gene is lessening worldwide, and should not be relied on alone for spotted wilt management. Thus, it is important to continue to monitor for emergence and spread of RB TSWV strains, and to search for sources of resistance to these strains, including new RNAi-based strategies for engineering tomato plants for resistance to tospovirus infection [2]. Regardless, the most effective and sustainable management of spotted wilt in tomato crops will require an IPM approach [5, 36].

## Supporting information

S1 Table Name, accession number, year of collection and location, host and strain of isolates of tomato spotted wilt virus including a wild-type (WT) and resistant-breaking (RB) isolates used in the present study. (DOCX)

S1 Fig Original uncropped and unadjusted images for all pictures of gels used in the Fig 1. (PDF)
