# Supplementary material for: An all-out assault on a dominant resistance gene: Local emergence, establishment, and spread of strains of tomato spotted wilt orthotospovirus (TSWV) that overcome Sw-5b-mediated resistance in fresh market and processing tomatoes in California
Source: PLoS One. 2024 Jul 10;19(7):e0305402. doi: 10.1371/journal.pone.0305402 (PMC11236122; doi:10.1371/journal.pone.0305402)
Supplement: S1 Table — (DOCX) [file pone.0305402.s001.docx]

**Table S1.** Name, accession number, year of collection and location, host and strain of isolates of tomato spotted wilt virus including a wild-type (WT) and resistant-breaking (RB) isolates used in the present study.

| **Name of isolates** | **Accession number** | **Year** | **Location** | **Host** | **Strain^a^** |
| --- | --- | --- | --- | --- | --- |
| 14_CA_Fresno_tomato | PP105562 | 2014 | USA: CA: Fresno | *Solanum lycopersicon* | WT-CPT |
| 15_CA_Fresno_Lettuce | PP105563 | 2015 | USA: CA: Fresno | *Lactuca sativa* | RB-FPT |
| 14_OB4_Mexico_tomato | PP105564 | 2014 | Mexico: Baja California Sur | *S. lycopersicon* | RB-YPT |
| 21_538_CA_San_Diego_tomato | PP105565 | 2021 | USA: CA: San Diego | *S. lycopersicon* | RB-FPT |
| 21_544_CA_San_Diego_tomato | PP105566 | 2021 | USA: CA: San Diego | *S. lycopersicon* | RB-FPT |
| 22_730_US_NJ_tomato | PP105567 | 2022 | USA: NJ: Cedarville | *S. lycopersicon* | RB-YPT |
| 23_24_CA_San_Diego_tomato | PP105568 | 2023 | USA: CA: San Diego | *S. lycopersicon* | RB-FPT |
| 23_39_CA_Colusa_tomato | PP105569 | 2023 | USA: CA: Colusa | *S. lycopersicon* | RB-YPT |
| 23_49_CA_Sutter_tomato | PP105570 | 2023 | USA: CA: Sutter | *S. lycopersicon* | RB-CPN |
| 23_51_CA_Sutter_tomato | PP105571 | 2023 | USA: CA: Sutter | *S. lycopersicon* | RB-YPT |
| 23_67_CA_Yolo_tomato | PP105572 | 2023 | USA: CA: Yolo | *S. lycopersicon* | RB-YPT |
| 23_136_CA_Colusa_tomato | PP105573 | 2023 | USA: CA: Colusa | *S. lycopersicon* | RB-CPN |
| 23_166_CA_San_Joaquin_tomato | PP105574 | 2023 | USA: CA: San Joaquin | *S. lycopersicon* | RB-YPT |
| 23_167_CA_San_Joaquin_tomato | PP105575 | 2023 | USA: CA: San Joaquin | *S. lycopersicon* | RB-YPT |
| 23_184_CA_Yolo_tomato | PP105576 | 2023 | USA: CA: Yolo | *S. lycopersicon* | RB-CPN |

^a^ Strain: WT, wild-type; RB, resistant-breaking; RB-FPT (C118F mutation); RB-YPT (C118Y mutation) and RB-CPN (T120N mutation).
