# Supplementary material for: An all-out assault on a dominant resistance gene: Local emergence, establishment, and spread of strains of tomato spotted wilt orthotospovirus (TSWV) that overcome Sw-5b-mediated resistance in fresh market and processing tomatoes in California
Source: PLoS One. 2024 Jul 10;19(7):e0305402. doi: 10.1371/journal.pone.0305402 (PMC11236122; doi:10.1371/journal.pone.0305402)

Fig. 1A RT-PCR for C118Y

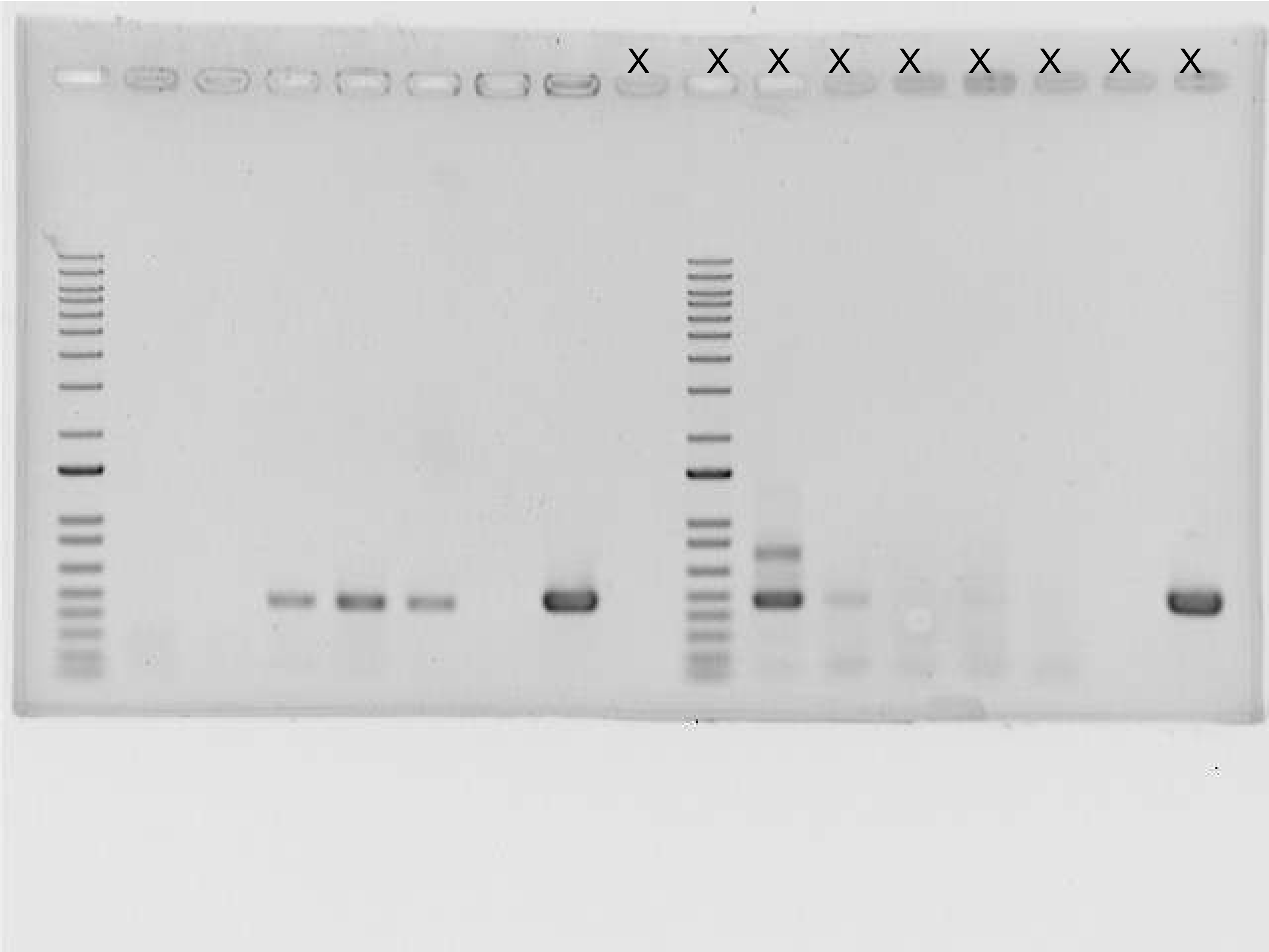

Fig. 1B RT-PCR for C118Y

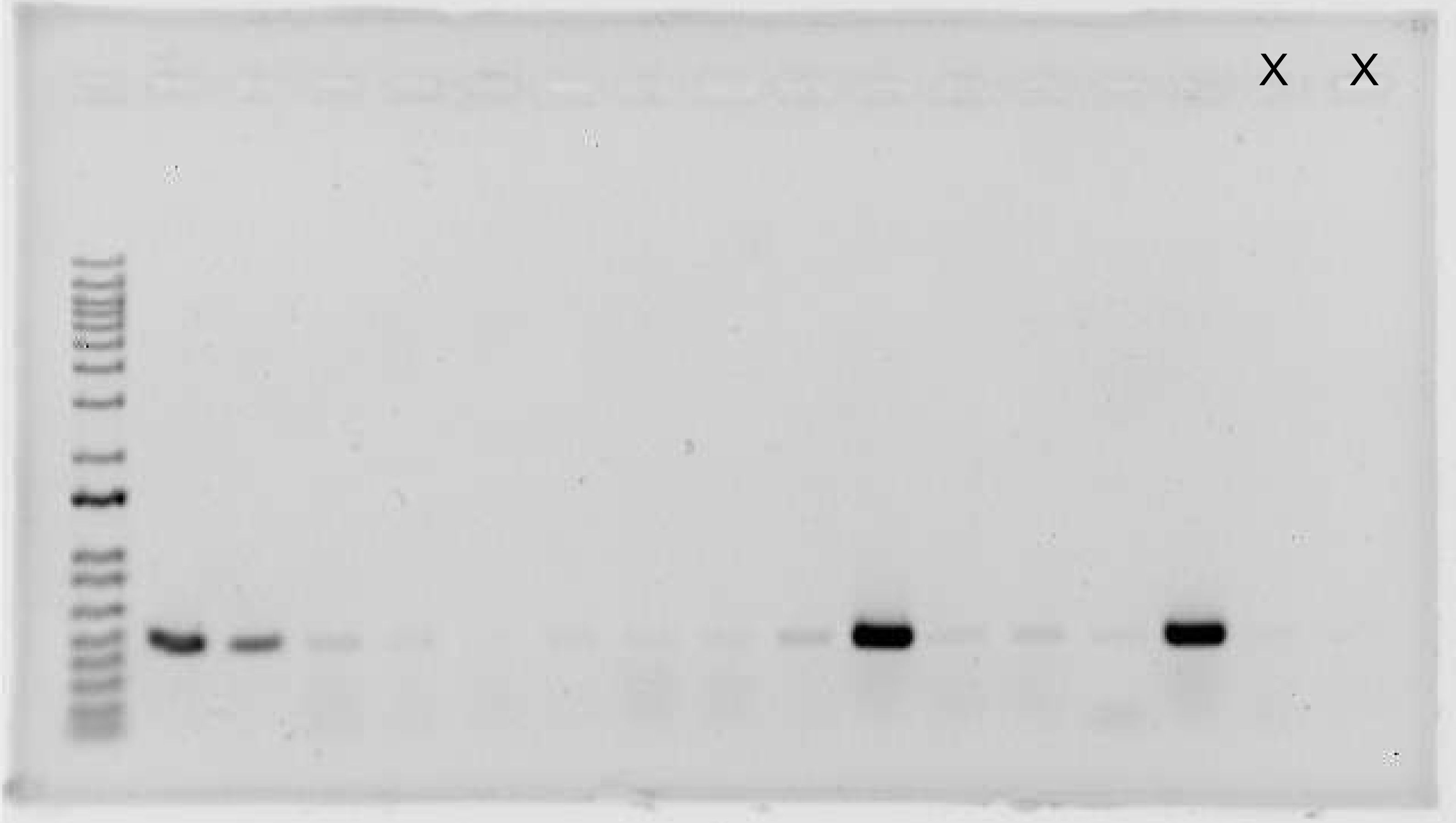

Fig 1B RT-PCR for NSm

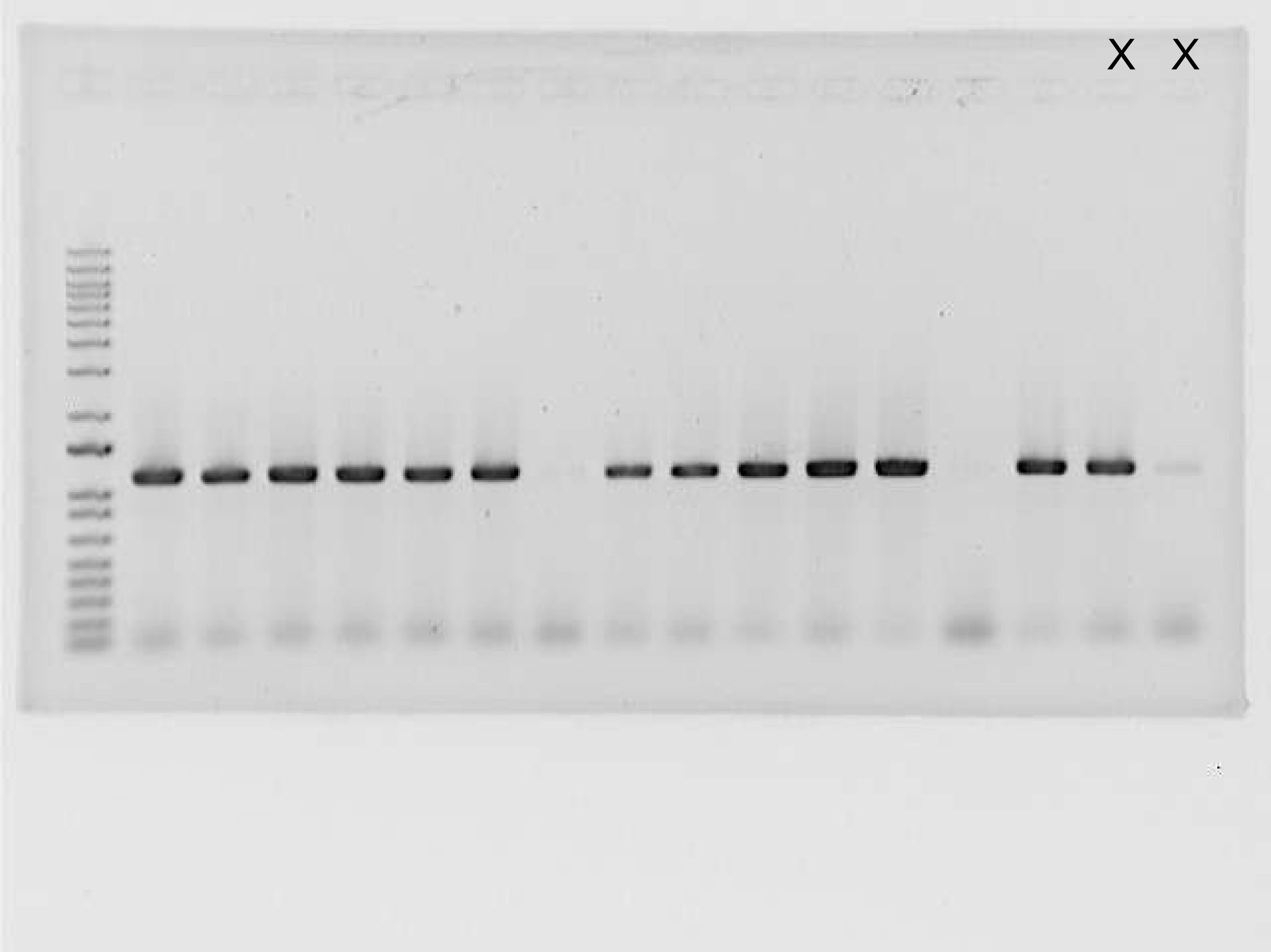

Fig. 1C RT-PCR for T120N

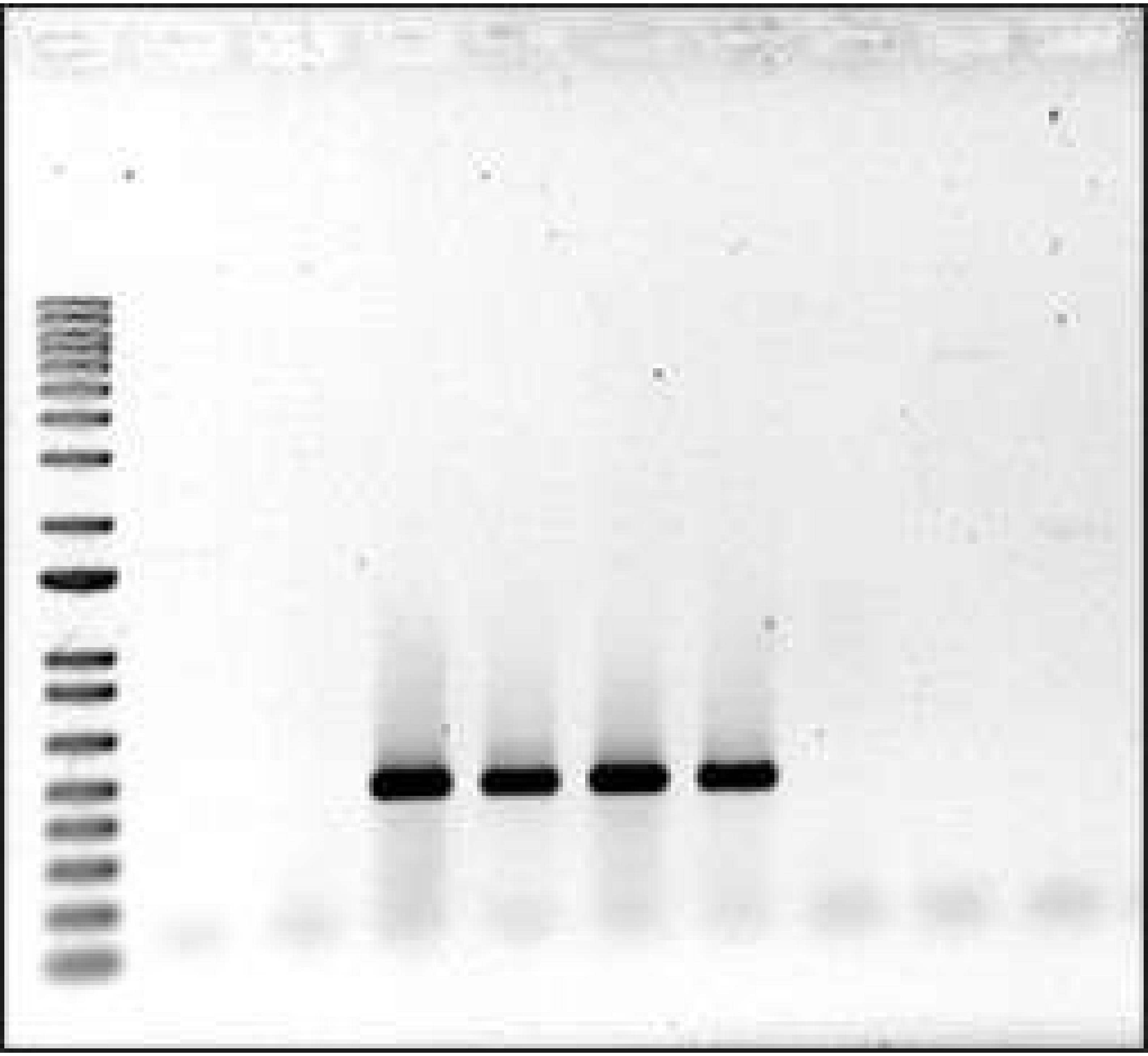

Fig. 1D RT-PCR for C118Y

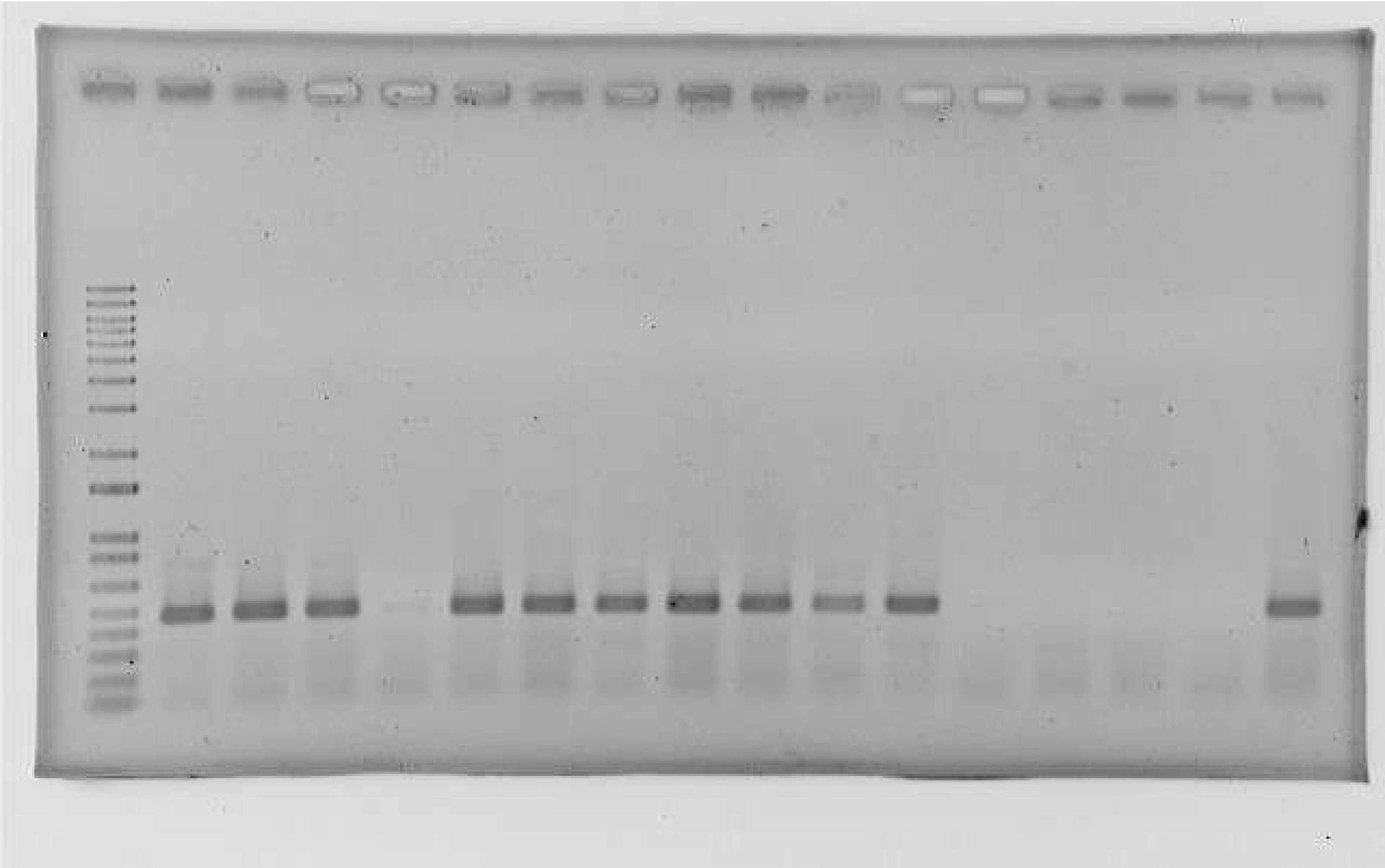

Fig. 1D RT-PCR for C118Y

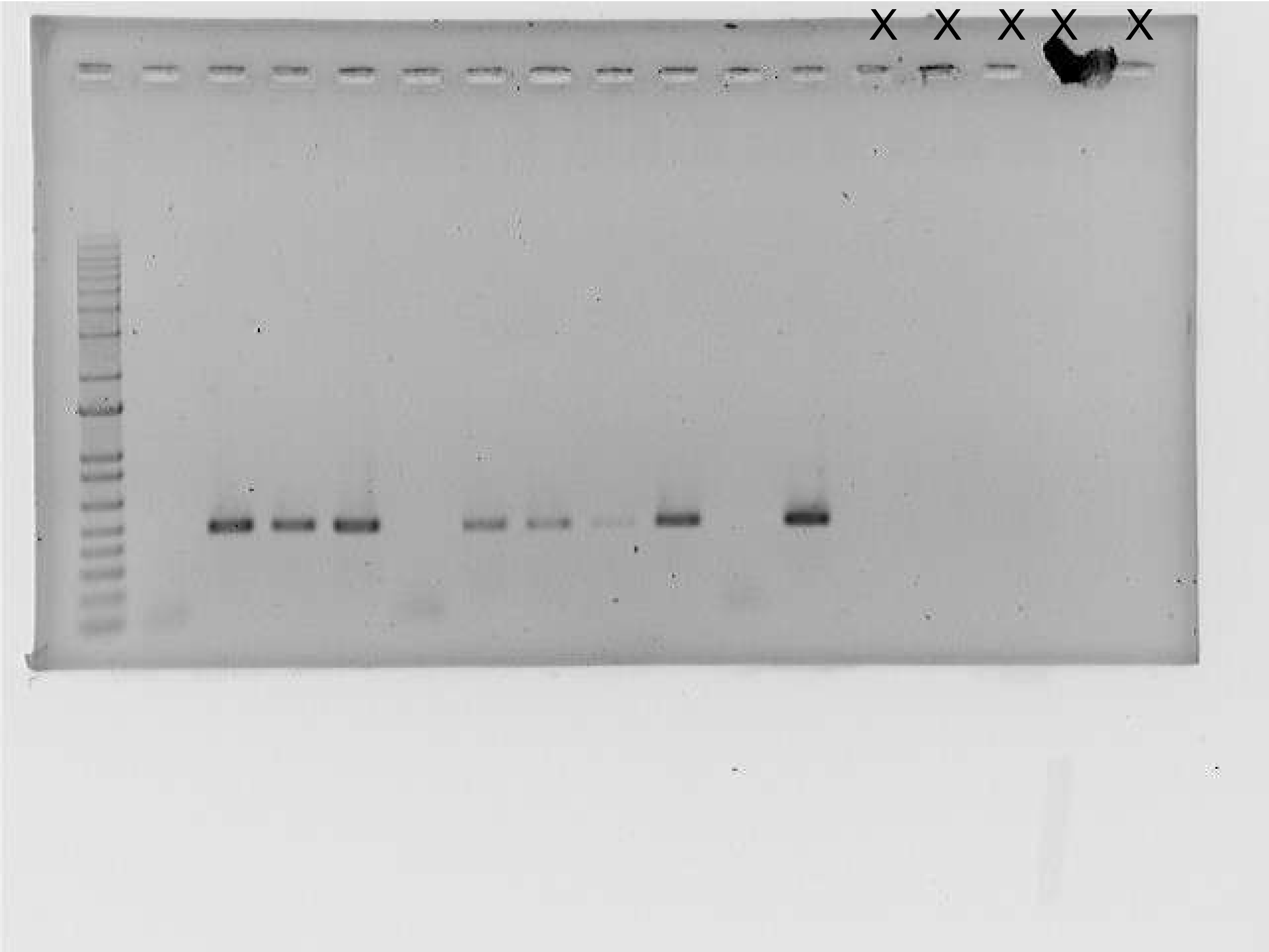

Fig. 1D RT-PCR for T120N

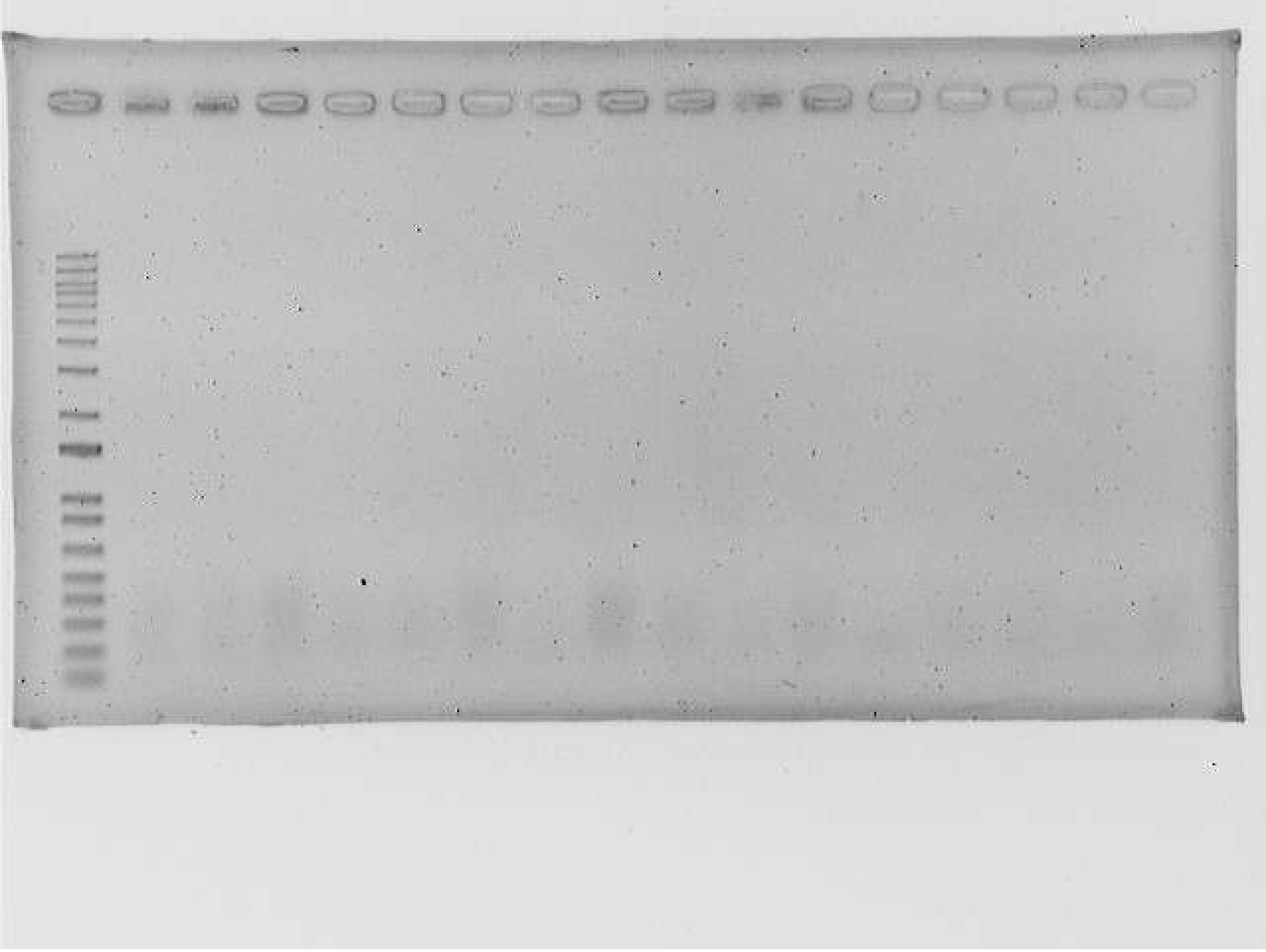

Fig. 1D RT-PCR for T120N

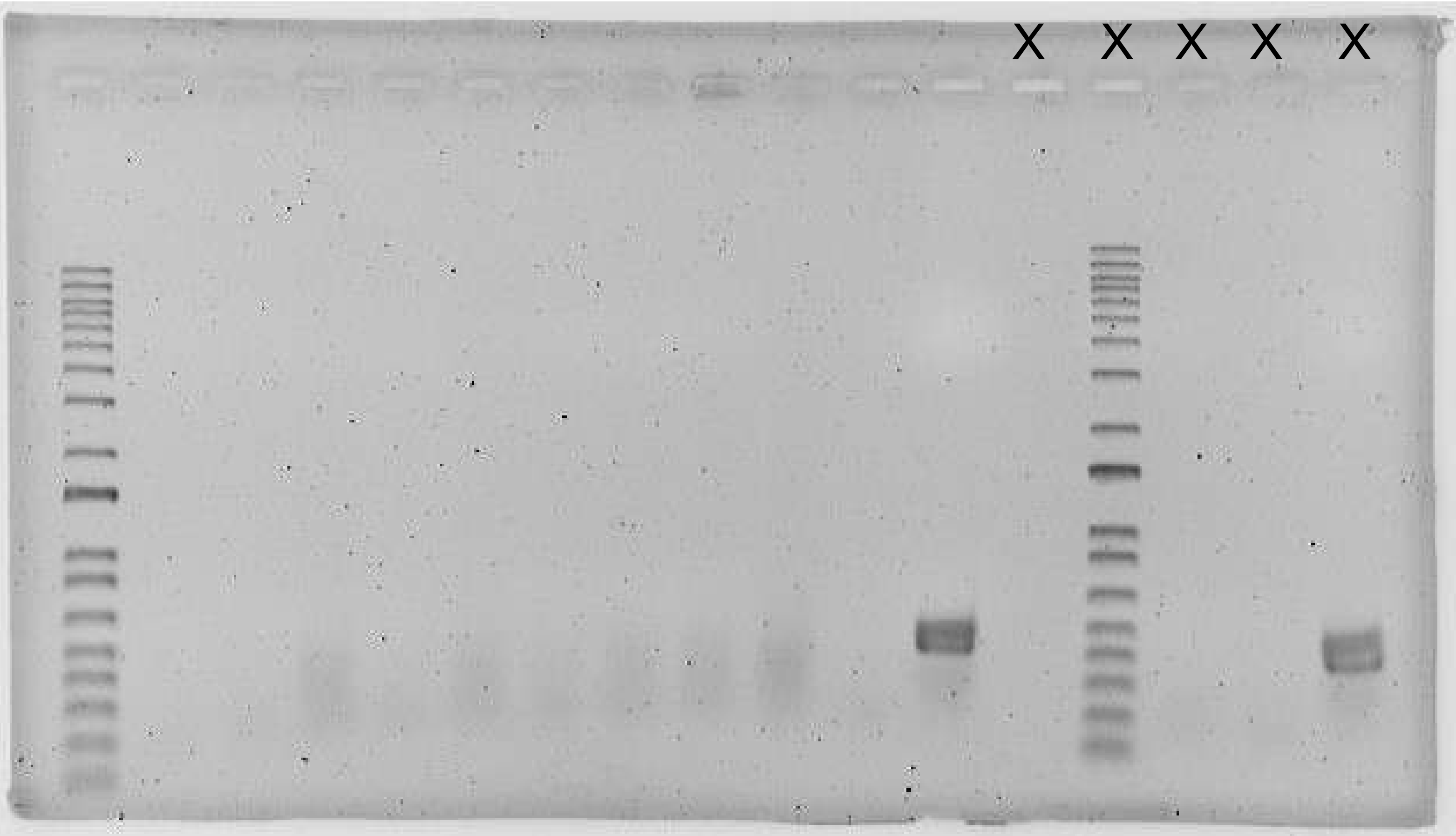

Supplement: S1 Fig — (PDF) [file pone.0305402.s002.pdf]
